# Multivalent scaffolds induce galectin-3 aggregation into nanoparticles

**DOI:** 10.3762/bjoc.10.162

**Published:** 2014-07-10

**Authors:** Candace K Goodman, Mark L Wolfenden, Pratima Nangia-Makker, Anna K Michel, Avraham Raz, Mary J Cloninger

**Affiliations:** 1 Department of Chemistry and Biochemistry, Montana State University, Bozeman, Montana 59717, USA; 2 The Departments of Oncology and Pathology, School of Medicine, Wayne State University, 110 East Warren Avenue, Detroit, Michigan 48201, USA

**Keywords:** dendrimers, galectin-3, glycodendrimers, multivalency, multivalent glycosylation, protein aggregation

## Abstract

Galectin-3 meditates cell surface glycoprotein clustering, cross linking, and lattice formation. In cancer biology, galectin-3 has been reported to play a role in aggregation processes that lead to tumor embolization and survival. Here, we show that lactose-functionalized dendrimers interact with galectin-3 in a multivalent fashion to form aggregates. The glycodendrimer–galectin aggregates were characterized by dynamic light scattering and fluorescence microscopy methodologies and were found to be discrete particles that increased in size as the dendrimer generation was increased. These results show that nucleated aggregation of galectin-3 can be regulated by the nucleating polymer and provide insights that improve the general understanding of the binding and function of sugar-binding proteins.

## Introduction

The role of multivalency in biology is well established, and examples of this phenomenon abound [1]. The ability of multivalency to enhance weak interactions has been shown in a variety of protein:carbohydrate systems using a wide assortment of scaffolds and carbohydrates [2]. As research with multivalent glycosystems advances, one important target for potential therapy and understanding is the galectin family of proteins [3]. Members of the galectin family share a common conserved sequence carbohydrate recognition domain (CRD) made of ~130 amino acids that are arranged in a folded beta-sheet structure and have an affinity for β-galactosides [4–7]. Galectin-3, one of the most studied members, is commonly up or down regulated in different cancers and is implicated in tumor formation and proliferation, apoptosis, angiogenesis, and B cell activation [8–10]. Galectin-3 has been reported to be involved in mechanisms that cluster cell surface glycoproteins [10–11], cross-link receptors [12], and form lattices and larger aggregates [13]. Structurally, galectin-3 is composed of one carbohydrate recognition domain and a collagen-like N-terminus tail [14].

The native quaternary structure of this unique galectin is a current topic of debate. Brewer et al. found that galectin-3 pentamers can be formed at high concentrations of protein [15], a noncovalent dimer and a monomer form of galectin-3 have also been reported [16–17]. Recent anisotropy binding measurements support two types of galectin-3 oligomerization, dominated by either N- or C-terminal interactions [18], and both N- and C-terminal domains are reported to be required for binding to targets such as lipopolysaccharide [19–20]. Galectin-3 can serve as a cellular docking site or a crosslinker for microorganisms binding to pathogens directly [20–21], and galectin-3 can act as a scaffold for the presentation of ligands such as lipopolysaccharides into an aggregate that stimulates cellular responses [19].

Binding affinities have been reported for a series of carbohydrate-based ligands to galectin-3, which binds to lactose significantly better than to galactose or to *N*-acetylgalactosamine but does not bind to mannose [22–24]. Both the glycan ligand and the topological display on the cell surface are required for high affinity, selective binding of galectins, as demonstrated in galectin binding studies with neuroblastoma cells [25].

Here, we demonstrate that glycodendrimers bearing lactose can be used to form large, discrete aggregates of galectin-3. Since, as noted above, glycan clustering and galectin-3-mediated aggregations have been demonstrated to be important for biological interactions ranging from bacterial invasion to cancer cellular responses, the development of systems such as glycodendrimers that can aggregate galectin-3 into nanoparticles in a highly controlled fashion is an important area of research. The study of galectin-3 binding and cluster formation by a series of glycodendrimers is a central step in the development of a synthetic multivalent antagonist that can intercept and influence galectin-3-mediated cellular processes and may be of clinical value as a non-cytotoxic drug and/or be developed for cancer imaging.

## Results and Discussion

### Synthesis and characterization of glycodendrimers

**Scheme 1:**
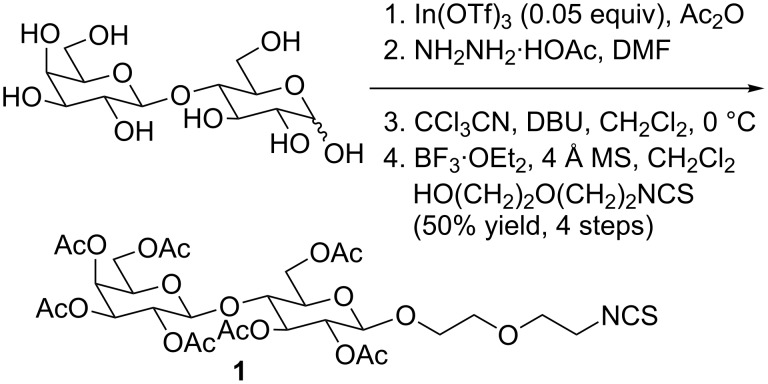
Synthesis of isothiocyanato-functionalized lactoside **1**.

Poly(amidoamine) (PAMAM) dendrimers are well-defined, water-soluble, symmetric scaffolds that contain a controlled and tuneable number of end groups. The number of end groups is specified by the dendrimer generation and approximately doubles for each subsequent generation [26]. These dendrimers are commercially available for generations zero, denoted G(0), to generation 10, denoted G(10). The amine termini can be functionalized with a variety of molecules, making these scaffolds an excellent choice for systematic studies of chemical and biological phenomena [27–28]. In this investigation, PAMAM dendrimers were functionalized using a methodology similar to previous literature [29]. Synthesis of β-lactoside derivative **1** was performed as shown in Scheme 1. Lewis acid facilitated glycosylation, which was directed by neighboring group participation of the 2-*O*-acetyl protecting group, afforded the desired anomers in good yields. The trichloroacetimidate intermediate was formed to enhance coupling.

**Scheme 2:**
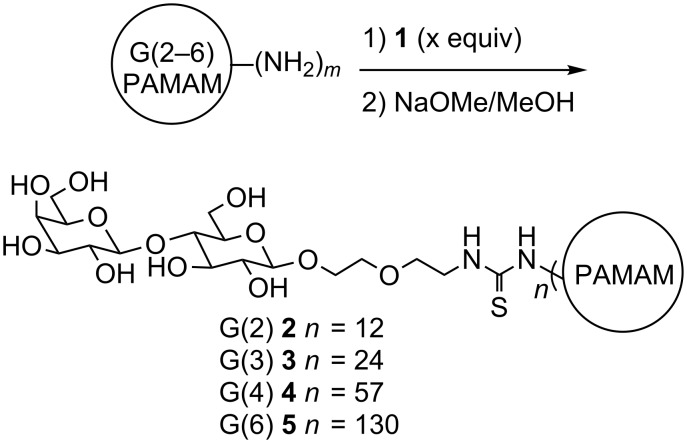
Synthesis of carbohydrate-functionalized PAMAM dendrimers. (Values for *m* and for x equivalents added are given in Supporting Information File 1.)

Syntheses of the carbohydrate-functionalized dendrimers were performed by addition of compound **1** as shown in Scheme 2. The functionalized dendrimers were characterized by MALDI–TOF–MS (matrix-assisted laser desorption time of flight mass spectrometry). The average numbers of sugars that were incorporated are shown in Scheme 2. The loadings were determined by both the changes in weight average molecular weight (*M*_w_) upon addition of **1** and the changes in *M*_w_ upon deacetylation, enabling characterization of the average number of sugars per dendrimer [30]. Additional characterization details (including ^1^H NMR spectra) are provided in Supporting Information File 1.

### Characterization of dendrimer/galectin-3 aggregates

Dynamic light scattering (DLS) was used to characterize the size and polydispersity of aggregate formation between lactose-functionalized dendrimers **2**, **3**, **4**, and **5**, with galectin-3. Three concentrations of glycodendrimers (11.5, 3.3 and 0.14 μM) were added to a constant concentration of galectin-3 (31 μM in PBS) to obtain a ratio of galectin-3 to glycodendrimer of 220:1, 9:1, or 3:1. These ratios were chosen so that results obtained from experiments using a large excess, a significant excess, and a slight excess of galectin-3 could be compared.

**Figure 1:**
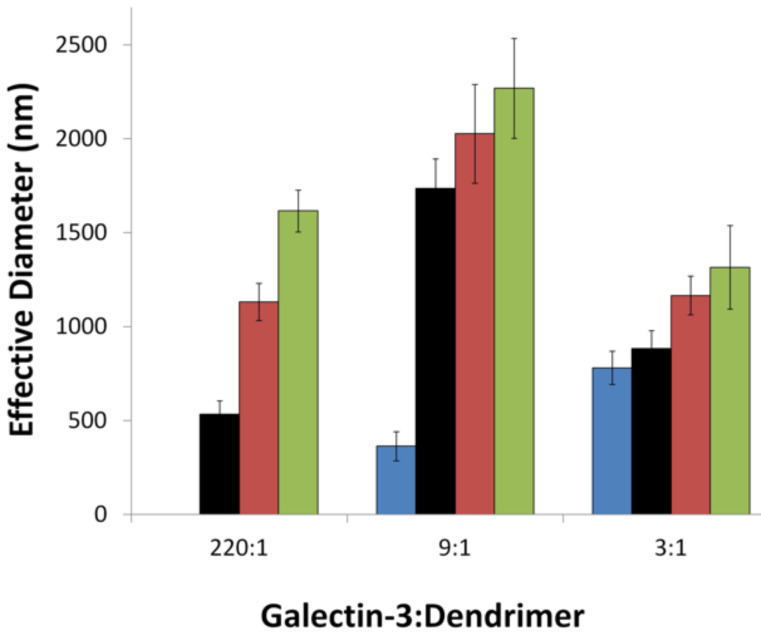
Effective diameter of galectin-3/glycodendrimer aggregates (DLS). Final concentration of galectin-3 31 μM; final concentration of glycodendrimers 0.14, 3.3, 11.5 μM for 220:1, 9:1, 3:1, respectively. **2** (blue), **3** (black), **4** (red) and **5** (green). Aggregate size was below the detection limit for **2** at 0.14 μM.

**Figure 2:**
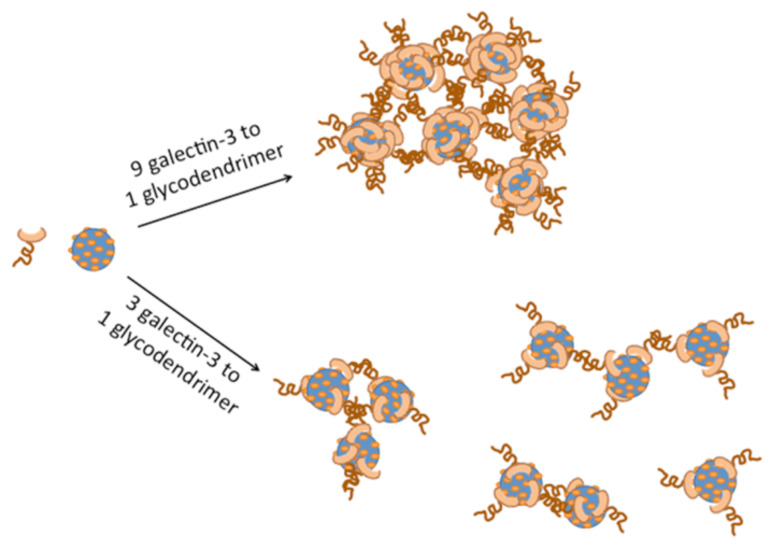
Schematic representation of galectin-3/glycodendrimer aggregates at varying stoichiometries.

Regardless of the amount of excess galectin-3 that was used, the size and polydispersity of the aggregates was shown to increase with increasing dendrimer generation (Figure 1 and Table 1). The largest aggregates were observed for the 9:1 ratio of galectin-3 to glycodendrimer, and smaller aggregates were formed when a large excess or a very small excess of galectin-3 was used. This trend is logical if the glycodendrimer is serving as the nucleating agent for galectin-3 aggregation. For example, when the concentration of galectin-3 is comparable to the concentration of glycodendrimer, then galectin-3 is presented with many different nucleating scaffolds, and smaller particles are formed. On the other hand, when galectin-3 is present in large excess, not as many nucleating sites can be incorporated into each aggregate, which causes the aggregates to be smaller. A schematic representation of glycodendrimer-mediated galectin-3 aggregation is shown in Figure 2. This trend was also observed in other systems. Ottaviani, et al. reported enhanced aggregation of amyloid peptides at low concentrations of maltose and maltotriose-functionalized poly(propylene imine) dendrimers and inhibition at high glycodendrimer concentrations [31]. Previous studies of asialofetuin/galectin-3 aggregation indicated that the glycoprotein ligand could serve to initiate aggregation, but carbohydrate binding was not required for all of the galectin-3 lectins that were involved in the interaction. Some galectin-3/galectin-3 interactions, in addition to carbohydrate/galectin-3 interactions, were proposed [18].

**Table 1:** Summary of aggregate characterization.

Compound	No. of particles	Mean diameter (FM, nm)	Avg. effective diameter (DLS, nm)

**2**	59	240 ± 50	not detectable
**3**	221	700 ± 290	560 ± 40
**4**	137	1070 ± 350	1180 ± 80
**5**	146	1790 ± 650	1620 ± 110

A series of control experiments indicate that aggregation is induced by the binding of galectin-3 to lactose on the dendrimers. No observable particles were detected upon addition of a mannose-functionalized G(4) dendrimer (Table S3 in Supporting Information File 1). Pre-incubation of galectin-3 with 1 mM lactose solution completely inhibited aggregate formation in the presence of glycodendrimers **3**, **4**, and **5** (Table S3 in Supporting Information File 1). Titration of a lactose solution into the solution of preformed aggregates did result in disassembly, but titration of an equivalent volume of PBS also resulted in disassembly (Table S3, Supporting Information File 1). Analysis of concentration effects and kinetics of aggregation and disaggregation are beyond the scope of this report but are under investigation. Experiments using **5** and truncated galectin-3, which has only the carbohydrate recognition domain without the N*-*terminal domain, did not result in aggregate formation (see Table S3, Supporting Information File 1). No aggregates were observed for dendrimers **2**–**5** in solution without addition of galectin-3, and no aggregates were observed for galectin-3 when glycodendrimers were absent from the solution. Taken together, these data support aggregate initiation as a response to specific carbohydrate binding interactions between lectin and glycodendrimer. They also reveal the significance of the N-terminal domain in formation of higher order aggregates.

**Figure 3:**
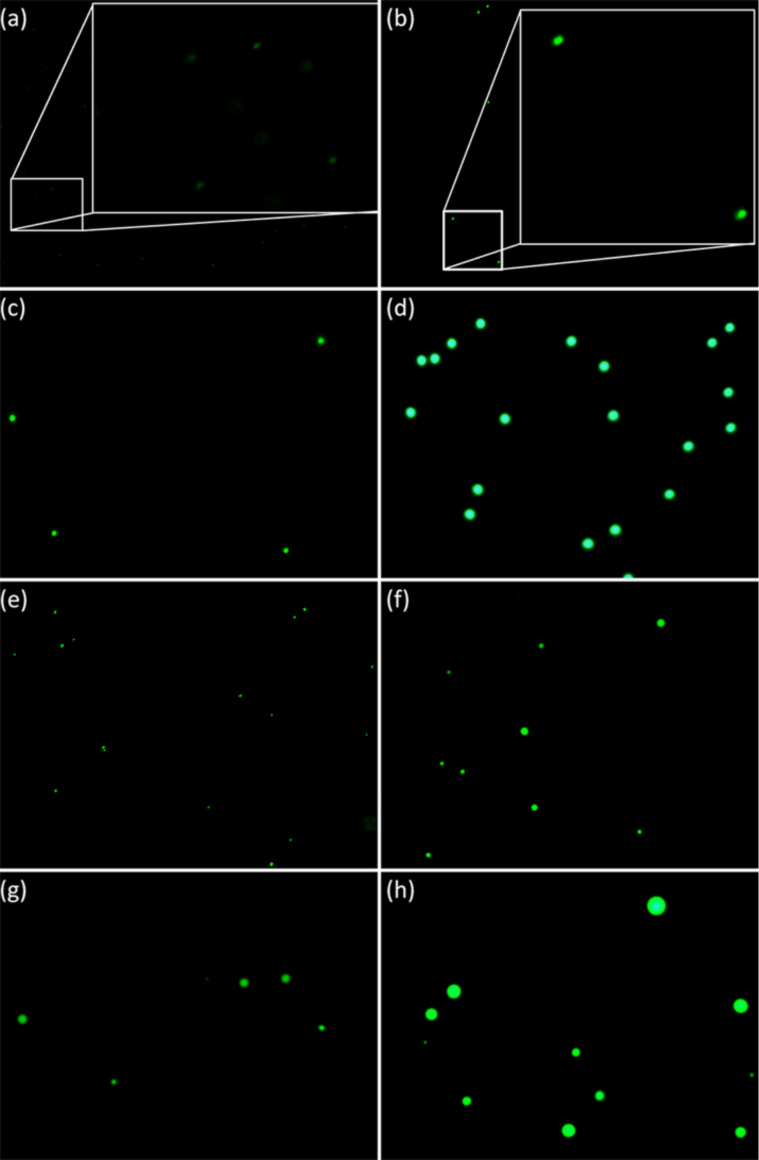
Fluorescence microscopy images of labelled particles. Microbead standards at similar exposure times are shown in (a)–(d); (a) 190 nm, inset is a 4× enlargement of selected area, (b) 520 nm, inset is a 4× enlargement of selected area, (c) 1020 nm and (d) 1900 nm. Aggregates formed after ca. 60 min incubation of Alexa 488 labelled galectin-3 (A488gal-3) with lactose-functionalized dendrimers are shown in (e)–(h); (e) A488gal-3 and **2**; (f) A488gal-3 and **3**; (g) A488gal-3 and **4**; (h) A488gal-3 and **5**. Exposure times of lectin–glycodendrimer aggregates are provided in Table S6, Supporting Information File 1.

**Figure 4:**
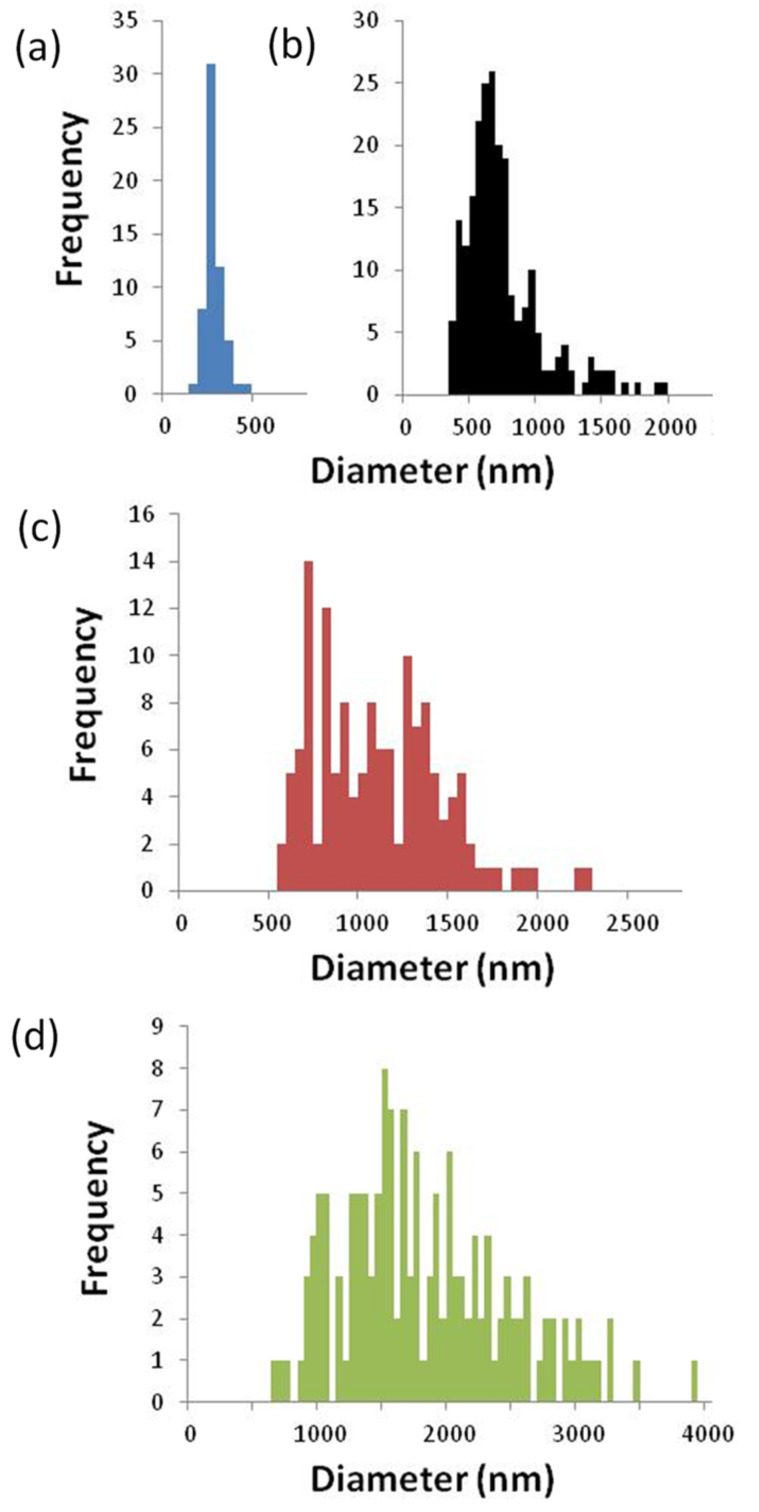
Aggregate diameter distribution of fluorescence microscopy images; 31 μM galectin-3 and 0.14 μM (a) **2**, (b) **3**, (c) **4** and (d) **5**.

The presence of these aggregates was confirmed by epifluorescence microscopy using galectin-3 fluorescently labelled with AlexaFluor 488 (A488gal-3, Figure 3). Following conjugation, the labelled galectin-3 was dialyzed against PBS to maintain identical conditions to DLS experiments. Size quantification using image analysis software (Pixcavator 6.0) and fluorescent microsphere standards (Dragon Green, Bangs Laboratories, Inc.) provided similar diameters as those obtained in DLS experiments (Figure 3, Table 1). The polydispersity calculated from the micrographs (Figure 4) was higher than that calculated by DLS, but this is likely due to sampling bias of DLS measurements as a result of attenuating the incident light (smaller particles that remain undetected in DLS were observed in microscopy images). For both methods, the trend of increasing size and polydispersity with increasing dendrimer generation was observed.

The aggregate size is remarkably large compared to galectin-3 and the dendrimers. The largest dendrimer used (generation 6) has a reported unfunctionalized diameter of 6.8 nm [32–33], and addition of the sugar adds about 4 nm to the overall diameter according to our DLS results with **5** (Table S3, Supporting Information File 1). The measured diameter of the CRD domain from the crystal structure of galectin-3 is roughly 3 nm [34]. The N-terminal domain consists of slightly fewer amino acids but is unstructured. Assuming the unstructured portion contributes about the same size or slightly more to the diameter of the protein as the CRD, the glycodendrimer complex would be expected to have a much smaller diameter than the observed values. Considering the large number of copies of dendrimer and protein that are required to form nanoparticle aggregates of the observed sizes, the aggregates are highly monodisperse. Although it has been previously determined using turbidity and precipitation assays that carbohydrate-functionalized dendrimers induce lectin aggregation, the consistent formation of large nanoparticles has to our knowledge not been previously identified and characterized.

The most likely explanation for the formation of large, monodisperse nanoparticle aggregates from galectin-3/glycodendrimer solutions is as follows. The glycodendrimer serves to nucleate the aggregation process through the specific binding of lactose into the carbohydrate binding site on galectin-3. Binding of the carbohydrate into the galectin-3 binding site must then be enabling protein–protein interactions. Some of these protein–protein interactions may occur because of intertwining of the N-terminal domains that are now in close proximity. However, protein–protein interactions using the carbohydrate recognition domains of galectin-3 after an initial carbohydrate binding event is entirely consistent with a recently proposed binding mechanism [18], and is also consistent with proposed models for scaffold-mediated nucleation of protein signalling complexes [35].

## Conclusion

In summary, β-lactoside functionalized PAMAM dendrimers **2**–**5** were synthesized and characterized. The presence of complex multivalent interactions between galectin-3 and lactose-functionalized dendrimers is indicated by the observation of large aggregates in DLS and epifluorescence experiments.

The large and relatively monodisperse nature of the glycodendrimer/galectin-3 aggregates that were formed (as determined by DLS and fluorescence microscopy) was dependent on both the dendrimer concentration and the generation. Third and fourth generation glycodendrimers formed smaller, more monodisperse aggregates, than sixth generation glycodendrimers. Aggregates formed at molar ratios of 9:1 galectin:glycodendrimer were largest while 220:1 and 3:1 ratios produced smaller complexes. The difference in aggregate sizes may relate to the size and shape complementarity between dendrimer and lectin or to the interplay of enthalpic and entropic contributions to aggregate formation as previously postulated [18,31,36]. Ongoing studies on the aggregate stoichiometry should provide valuable insight on this matter.

Overall, the results presented here indicate that clustering and aggregation events should be considered in addition to carbohydrate binding affinity for galectin-3, and also for other biological processes that are mediated by multivalent carbohydrate–protein interactions.

## Experimental

### General experimental methods

General reagents were purchased from Acros and Aldrich Chemical Companies. PAMAM dendrimers were purchased from Dendritech. Fluorescent microbead standards were purchased from Bangs Laboratories, Inc.. Dichloromethane was purified on basic alumina; other solvents were used as received. Silica gel (32–63 μm “40 micron flash”) for flash column chromatography purification was purchased from Scientific Adsorbants Incorporated.

#### 5-Isothiocyanato-3-oxapentyl 2,3,4,6-tetra-*O*-acetyl-β-D-galactopyranosyl-[1→4]-2,3,6-tri-*O*-acetyl-β-D-glucopyranoside (**1**)

2,3,4,6-Tetra-*O*-acetyl-β-D-galactopyranose-[1→4]-1,2,3,6-tetra-*O*-acetyl-β-D-glucopyranose (4.4 g, 6.4 mmol) was dissolved in dry DMF (20 mL). Hydrazine acetate (0.77 g, 8.4 mmol) was added and the reaction mixture was heated to 55 °C for 1 h. The mixture was diluted into CH_2_Cl_2_ (20 mL) and washed with brine (2 × 10 mL) and water (2 × 10 mL), dried over MgSO_4_, filtered and the solvent was removed in vacuo. The residual product was added to a solution of trichloroacetonitrile (3.34 g, 23.1 mmol) in CH_2_Cl_2_ (20 mL). After cooling the mixture in an ice bath, DBU (60 mg, 0.32 mmol) was added drop-wise and the mixture was stirred for 3 h. The reaction mixture was dissolved in CH_2_Cl_2_ (30 mL), and the organic layer was washed with brine (2 × 10 mL) and water (2 × 10 mL), dried over MgSO_4_, filtered and the solvent was removed in vacuo. The residual product was taken up in CH_2_Cl_2_ (50 mL) with 2-(2-isothiocyantoethoxy)ethanol (0.6 g, 4 mmol) and 4 Å molecular sieves. BF_3_OEt_2_ (0.6 g, 4 mmol) was added to the mixture over 30 min at 0 °C, and the reaction mixture was let stir and warmed to room temperature over 2 h. The solvent was removed and the residue was taken up in ethyl acetate (50 mL), washed with saturated aqueous NaHCO_3_ solution (2 × 20 mL), brine (2 × 20 mL), and water (1 × 20 mL), dried over MgSO_4_, filtered and the solvent was removed in vacuo. The oily residue was purified by silica gel column chromatography with a 60:40 ethyl acetate/hexanes eluent, followed by a 20:1 ethyl acetate/MeOH eluent to yield 2.6 g of product. ^1^H NMR (300 MHz, CDCl_3_) δ 5.33 (d, *J* = 3.1 Hz, 1H, H4’), 5.20 (app t, *J* = 9.3 Hz, 1H, H3), 5.09 (dd, *J* = 8.1 and 10.1 Hz, 1H, H2’), 4.93 (m, 2H, H2 and H3’), 4.50 (m, 3H, H1, H1’ and H6), 4.06 (m, 4H), 3.89 (m, 6H), 3.78 (m, 3H), 3.63 (m, 6H), 2.13 (s, 3H), 2.10 (s, 3H), 2.04 (s, 3H), 2.02 (m, 9H), 1.95 (s, 3H). As reported [37].

#### General procedure for the synthesis of acetyl-protected lactose-functionalized dendrimers

An aqueous solution of amine terminated G(4)-PAMAM dendrimer (2.48 g of a 17% w/w solution in water, 421 mg, 31.2 μmol) was lyophilized to leave a foamy residue. 7.02 mL of DMSO was added to this residue to give a 60 mg/mL solution of the dendrimer. 0.47 mL of a 300 mM solution of **1** (184 mg, 141 μmol) was added to 0.5 mL of the 60 mg/mL G(4) PAMAM dendrimer (30 mg, 2.20 μmol) solution. The mixture was stirred for 8 h at which point a 75 μL aliquot was collected and lyophilized for MALDI–TOF and NMR analysis. The remainder of the reaction mixture was lyophilized and subjected to the deacetylation procedure. This procedure for carbohydrate functionalization of dendrimers was performed in a manner similar to our previously described procedure [29]. Amounts used in the syntheses of **2–5** are provided in Table S1, Supporting Information File 1. Characterization data for acetylated precursors of **2–5** are provided in Supporting Information File 1.

#### General procedure for deacetylation to afford lactose-functionalized dendrimers

To the lyophilized solid per-*O*-acetylated dendrimers, 1 mL of 1:1 water/methanol was added, at which point the dendrimer became a white precipitate. To this mixture was added 0.2 equiv of NaOMe (0.8 M in MeOH) for each peripheral carbohydrate, and let stir for 3 h. If, at this time, the mixture had not become a clear solution a further 0.2 equiv of NaOMe (0.8 M in MeOH) was added and this step was repeated until the mixture became a clear and colourless solution. Aqueous HCl (0.1 M) was then added slowly until the pH was ~7. This neutralized solution was placed in a dialysis membrane (MW cutoff 3500 Da) and dialyzed in 1 L of deionized water for 8 h. The water was changed and let stand for a further 8 h twice more. The remaining liquid in the membrane was frozen and lyophilized to give a white fluffy solid. This procedure for deacetylation was performed in a manner similar to our previously described procedure [29]. Characterization data for dendrimers is provided in Supporting Information File 1.

#### NMR spectroscopy

^1^H NMR spectra were recorded on Bruker DPX 300 (300 MHz) and Bruker DPX-500 (500 MHz) spectrometers. Chemical shifts are reported in ppm from tetramethylsilane with the residual protic solvent resonance as the internal standard (chloroform: δ 7.25 ppm; dimethyl sulfoxide: δ 2.50 ppm). Data are reported as follows: chemical shift, multiplicity (s = singlet, bs = broad singlet, d = doublet, t = triplet, q = quartet, p = pentet, m = multiplet, app = apparent), integration, coupling constants (in Hz) and assignments. Sample NMR spectra are provided in Figures S2 through S6, Supporting Information File 1.

#### MALDI–TOF mass spectrometry

MALDI mass spectra were acquired using a Bruker Biflex-III time-of-flight mass spectrometer. Spectra of all-functionalized dendrimers were obtained using a *trans*-3-indolacrylic acid matrix with a matrix:analyte ratio of 3000:1 or 1000:1. Bovine serum albumin (*M*_w_ 66,431 g/mol), cytochrome C (*M*_w_ 12,361 g/mol), and trypsinogen (*M*_w_ 23,982 g/mol) were used as external standards. An aliquot corresponding to 12–15 pmol of the analyte was deposited on the laser target. Positive ion mass spectra were acquired in linear mode and the ions were generated by using a nitrogen laser (337 nm) pulsed at 3 Hz with a pulse width of 3 nanoseconds. Ions were accelerated at 19,000–20,000 volts and amplified using a discrete dynode multiplier. Spectra (100 to 200) were summed into a LeCroy LSA1000 high-speed signal digitizer. All data processing was performed using Bruker XMass/XTOF V 5.0.2. Molecular mass data and polydispersities (PDI) of the broad peaks were calculated by using the Polymer Module included in the software package. The peaks were analysed using the continuous mode. *M*_w_ values for **2**–**5** are provided in Table S2, Supporting Information File 1.

#### Dynamic light scattering (DLS)

DLS measurements were acquired with the Brookhaven 90Plus Particle Size Analyzer equipped with a 15 mW solid state, 633 nm laser and upgraded APD detector. Scattered light was detected at 90° incidence and optimized to a count rate of 200–400 kilocounts per second (kcps) through adjustment of a neutral density filter prior to the sample chamber. The intensity was maximized for samples producing less than 200 kcps. Temperature control was stabilized at 25 °C, and each sample was scanned for 5 min (3 min for control samples). Autocorrelation curves were analyzed via the provided software using the method of cumulants (quadratic fit) unless otherwise noted. This provided the effective diameter and relative variance reported below. For dust-free, relatively monodispersed samples, this analysis provided results similar to NNLS and CONTIN algorithms. Protocols for preparation of solutions and a brief discussion of the theory for DLS are provided in Supporting Information File 1. Representative fitted data is shown in Supporting Information Fle 1, Figure S7 for **4** and **5**.

#### Fluorescence microscopy

Fluorescence images were captured on either a Nikon Eclipse TE2000-U with a 60× oil immersion objective lens (**2**, **3**, **4**) or Olympus BX-61 with a 100× oil immersion objective (**3**, **4**, **5**). Exposure time was optimized for each sample and 20–30 images were taken. These images were combined using Gimp 2 image manipulation software. Fluorescent microsphere standards (190, 520, 1020, and 1900 nm, Bangs Laboratories, Inc.) were used to calibrate the measured particle perimeter (pixels) to particle diameter (nm) for each exposure setting (Supporting Information File 1, Figure S8, equations in Table S4). Diameters of standards were verified with DLS (Table S5, Supporting Information File 1). The imaged particle perimeters were determined through Pixcavator Image Analysis software (Intelligent Perception). The y-intercept of each calibration curve represents the lower detection limit for the given exposure time. Protocols for preparation of samples for fluorescence microscopy are provided in Supporting Information File 1.

## Supporting Information

File 1 Amounts of reagents used in glycodendrimer syntheses; characterization data for glycodendrimers; sample calculations; detailed protocols for galectin-3 isolation and solution and sample preparations; sample NMR spectra; characterization data for glycodendrimer aggregates.
