# Supplementary material for: Multivalent scaffolds induce galectin-3 aggregation into nanoparticles
Source: Beilstein J Org Chem. 2014 Jul 10;10:1570–7. doi: 10.3762/bjoc.10.162 (PMC4142985; doi:10.3762/bjoc.10.162)
Supplement: File 1 — Amounts of reagents used in glycodendrimer syntheses; characterization data for glycodendrimers; sample calculations; detailed protocols for galectin-3 isolation and solution and sample preparations; sample NMR spectra; characterization data for glycodendrimer aggregates. [file Beilstein_J_Org_Chem-10-1570-s001.pdf]

Supporting Information

for

Multivalent scaffolds induce galectin-3 aggregation into nanoparticles

Candace K. Goodman[1], Mark L. Wolfenden[1], Pratima Nangia-Makker[2], Anna K. Michel[1], Avraham Raz[2], and Mary J. Cloninger*[1]

Address: Department of Chemistry and Biochemistry, Montana State University, Bozeman, Montana 59717, USA and [2]The Departments of Oncology and Pathology, School of Medicine, Wayne State University, 110 East Warren Avenue, Detroit, Michigan 48201, USA

Email: Mary J. Cloninger - mcloninger@chemistry.montana.edu

*Corresponding author

Amounts of reagents used in glycodendrimer syntheses; characterization data for glycodendrimers; sample calculations; detailed protocols for galectin-3 isolation and solution and sample preparations; sample NMR spectra; characterization data for glycodendrimer aggregates

Table of Contents

Page

Synthesis and characterization of lactose-functionalized dendrimers S2–S3
Expression and purification of galectin-3 ....S4-S5
Dynamic light scattering theory, sample preparation and results S5–S8
Fluorescence microscopy image calibration and sample preparation S9–S10
Sample NMR spectra S11–S15
Sample DLS correlation function S16
Fluorescence microscopy calibration curves and exposure times.................. S17
References.. S18

Synthesis and characterization of lactose-functionalized dendrimers

Amounts used in the syntheses of **2–5** are provided in Table S1. The number of amino endgroups prior to functionalization, as determined by MALDI–TOF–MS and denoted "m" in Scheme 2, was 16, 32, 60, and 212 for G(2), G(3), G(4), and G(6)-PAMAMs, respectively. Characterization data for acetylated precursors and final products **2–5** (deacetylated dendrimers) are provided below.

Table S1: Amounts used in synthesis of compounds **2–5**.

Compound Number	PAMAM Generation	PAMAM (μmol)	Lactose (μmol) (x^a)
**2**	2	9.2	157.0
**3**	3	4.4	140.8
**4**	4	2.2	140.8
**5**	6	0.5	120.0

[a]Values for x (as shown in Scheme 2 of the main manuscript) derive directly from the μmol values shown here.

Acetylated dendrimers.

Although these dendrimers are precursors of **2–5**, the product numbers are used for simplicity of record keeping.

2: ^{1}H NMR (500 MHz, DMSO-D$_6$) δ 7.98 (1H, bs), 7.80 (0.9H, bs), 7.50 (1.1H, bs), 5.20-5.17 (0.8H, m), 5.15-5.06 (1.3H, m), 4.83-4.75 (1.0H, m), 4.74-4.68 (1.1H, m), 4.67-4.60 (0.6H, m), 4.30-4.22 (0.8H, m), 4.20-4.15 (0.9H, m), 4.09-3.93 (3.0H, m), 3.81-3.68 (2.3H, m), 3.62-3.31 (7.5H, m), 3.13 (2.3H, bs), 3.04 (2.0H, bs), 2.62 (4.5H, bs), 2.47 (1.7H, s), 2.39 (2.1H, bs), 2.16 (3.6H, bs), 2.06 (2.4H, s), 2.04 (2.6H, s), 1.99-1.95 (5.2H, m), 1.94-1.91 (3.1H, m), 1.86 (2.1H, s) ppm. MALDI-TOF (pos) m/z 11290.

3: ^{1}H NMR (500 MHz, DMSO-D$_6$) δ 7.97 (1H, bs), 7.78 (0.9H, bs), 7.47 (1.5H, bs), 5.17 (1.3H, s), 5.11 (2.4H, m), 4.79 (1.5H, app t, J = 9.0 Hz), 4.69 (2.2H, m), 4.62 (1.1H, J = 9.0Hz), 4.25 (1.2H, d, J = 11.3Hz), 4.16 (1.4H, m), 3.97 (4.3H, m), 3.74 (3.7H, m), 3.56 (2.5H, bs), 3.12 (2.7H, bs), 3.04 (2.0H, bs), 2.64 (5.7H, bs), 2.16 (4.4H, bs), 2.04 (4.3H, s), 2.02 (4.8H, s), 1.95 (8.9H, s), 1.92 (7.3H, s), 1.84 (3.9H, s) ppm. MALDI-TOF (pos) m/z 24400.

4: ^{1}H NMR (500 MHz, DMSO-D$_6$) δ 7.95 (1H, bs), 7.78 (0.8H, bs), 7.47 (1.9H, bs), 5.17 (1.7H, s), 5.11 (2.8H, m), 4.79 (1.7H, app t, J = 9.0 Hz), 4.69 (2.5H, m), 4.62 (1.2H, J = 9.0Hz), 4.25 (1.4H, d, J = 10.8Hz), 4.16 (1.6H, m), 3.97 (4.9H, m), 3.74 (4.4H, m), 3.40-3.56 (10H, m), 3.12 (2.7H, bs), 3.04 (1.4H, bs), 2.64 (4.9H, bs), 2.16 (3.8H, bs), 2.04 (5.2H, s), 2.02 (4.7H, s), 1.95 (10.5H, s), 1.92 (7.4H, s), 1.84 (4.6H, s) ppm. MALDI-TOF (pos) m/z 54500.

5: ^{1}H NMR (500 MHz, DMSO-D$_6$) δ 7.96 (1H, bs), 7.76 (0.7H, bs), 7.47 (1.5H, bs), 5.17 (1H, s), 5.11 (1.8H, m), 4.79 (1.2H, m), 4.69 (1.5H, m), 4.62 (0.9H, m), 4.25 (0.9H, m), 4.16 (1H, m), 3.97 (3.2H, m), 3.74 (2.9H, m), 3.56 (1.6H, bs), 3.04-3.12 (3.8H, m), 2.64 (2.4H, bs), 2.16 (3.3H, bs), 2.04 (3H, s), 2.02 (3.4H, s), 1.95 (7.1H, s), 1.92 (4.9H, s), 1.84 (3.1H, s) ppm. . MALDI-TOF (pos) m/z 147000.

Deacetylated dendrimers **2–5**

2: ^{1}H NMR (500 MHz, DMSO-D$_6$) δ 8.16 (1H, bs), 7.57 (1.2H, m), 5.23-4.92 (1.0H, m), 4.89-4.39 (3.6H, m), 4.33-4.25 (0.3H, m), 4.24-4.08 (1.1, m), 3.90-3.76 (1.0H, m), 3.74-3.07 (solvent obstructed, m), 23.03-2.85 (3.0H, m), 2.46 (2.3H, m) ppm. MALDI-TOF (pos) m/z 9230.

3: ^{1}H NMR (500 MHz, DMSO-D$_6$) δ 8.02 (bs, 1H), 7.82 (bs 0.9H), 7.54 (bs, 1.4H), 5.12 (bm, 1.8H), 4.64 (m, 3.7H), 4.33 (m, 2.1H), 3.87 (d, J = 4.8Hz, 1.4H), 3.74 (m, 1.7H), 3.47-3.62 (m, 18H), 3.17 (bs, 2.6H), 3.08 (bs, 1.7H), 3.03 (m, 1.3H), 2.66 (bs, 3.7H), 2.43 (bs, 1.9H), 2.20 (bs, 3.6H), 1.89 (s, 0.4H), 1.80 (s, 0.2H) ppm. MALDI-TOF (pos) m/z 15000.

4: ^{1}H NMR (500 MHz, DMSO-D$_6$) δ 8.03 (bs, 1H), 7.86 (bs 0.8H), 7.52 (bs, 1.7H), 5.22 (bs, 1.0H), 5.12 (bm, 1.1H), 4.82 (bs 1.1H), 4.72 (m, 2.1H), 4.55 (m, 1.9H), 4.22 (m, 2.1H), 3.87 (d, 1.4H), 3.74 (m, 1.8H), 3.47-3.62 (m, 34H), 3.17 (bs, 2.2H), 3.03 (bs, 1.2H), 2.70 (bs, 3.1H), 2.23 (bs, 3.2H) ppm. MALDI-TOF (pos) m/z 31200.

5: ^{1}H NMR (500 MHz, DMSO-D$_6$) δ 8.02 (bs, 1H), 7.81 (bs 0.9H), 7.53 (bs, 1.3H), 5.22 (bs, 0.8H), 5.12 (bs, 0.8H), 4.64 (m, 3.7H), 4.22 (m, 1.7H), 3.87 (m, 1.2H), 3.74 (bs, 1.4H), 3.47-3.62 (m, 28H), 3.17 (bs, 1.8H), 3.08 (bs, 2.4H), 2.66 (bs, 2.7H), 2.43 (bs, 1.3H), 2.20 (bs, 3.0H) ppm. MALDI-TOF (pos) m/z 100000.

Table S2: MALDI-TOF MS data for dendrimers **2–5.**

Compound number	M_w of PAMAM starting material	M_w after addition of **1**	M_w after deacylation
**2**	3220	11290	9230
**3**	6800	24400	15000
**4**	13500	54500	31200
**5**	51000	147000	100000

Expression and purification of galectin-3

Following previously published methods [1], an 80 mL culture of *E. coli* transformed with pGEX-6p-galectin-3 was grown overnight (37 °C, 250 rpm) in YT/AMP media then transferred to 1 L YT/AMP media and grown until the OD_{600} reached 1.2 (1–2 h). At this point, 1 mL of 100 mM IPTG was added and incubation continued for another 4–5 h. Bacteria were harvested by centrifuging for 15 min at 4000 rpm. Resuspended pellets (20% w/w in 1 x PBS) were microfluidized then centrifuged at 9000 rpm for 10 min. The supernatant was incubated (1 h, 4 °C) with 0.25 mL glutathione sepharose (GS) 4B beads (50% w/w in PBS, GE Healthcare) per 12 mL supernatant. These were centrifuged (2000 rpm, 5 min) and washed 2–3 times with 3 mL 1 x PBS then once with 1 mL cleavage buffer (50 mM Tris-HCl pH 7, 150 mM NaCl, 1 mM EDTA, 1 mM DTT). Overnight incubation in 0.920 mL cleavage buffer and 0.080 mL PreScission Protease (GE Healthcare) yielded the purified galectin-3. The beads were washed 2 x with 1 x PBS to remove all galectin-3. These were combined and dialyzed against 1 x PBS (2–6 h, on ice, switching buffer 2–3 times). The absorbance at 280 nm relative to buffer (1 x PBS) was used to dilute the combined samples to a final concentration of 1.3 mg/mL (A_{280} = 0.81) as previously reported [1,2]. Purity of the galectin-3 product was verified by running a 15% SDS-PAGE gel on protein samples. Figure S1 shows the purified galectin-3 removed from the GS beads, the two additional GS bead washes and combined, dialyzed product (1.3 mg/mL). A single band is observed between 25 and 30 kDa, as expected for galectin-3 (Figure S1).

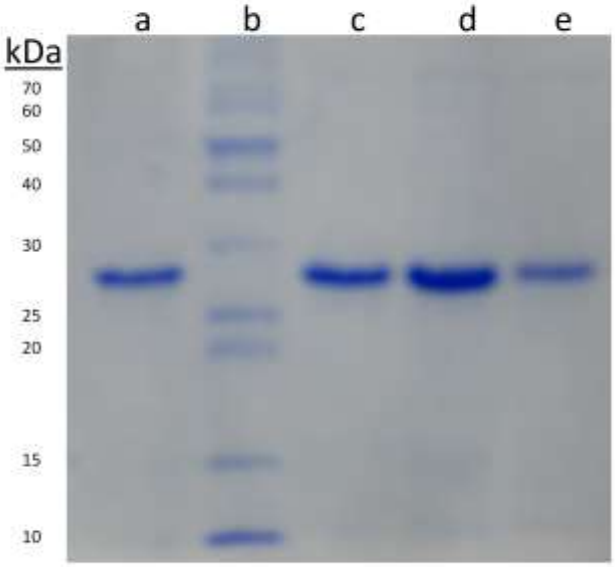

Figure S1: SDS-PAGE of galectin-3 product. (a) Galectin-3 sample after dialysis and dilution. (b) Molecular weight ladder. (c) 1st wash of GS beads following protease cleavage. (d) Eluent obtained after protease cleavage. (e) 2nd PBS wash of GS beads following protease cleavage of galectin-3.

Dynamic light scattering theory, sample preparation and results

Theory: The autocorrelation function ($g(t)$) is fit assuming particles are spherical and can be generally defined by the equation below:

$$g(t) = \int G(\Gamma) e^{-\Gamma t} d\Gamma \qquad (1)$$

where $G(\Gamma)$ is the distribution function, t refers to the time delay between measurements and Γ is the product of the translational diffusion coefficient (D) and the scattering efficiency of the molecule (q). Mathematically, Γ is defined as:

$$\Gamma = Dq^2 = \left(\frac{k_B T}{3\pi\eta d}\right)\left(\frac{2\pi n}{\lambda} 2\sin\left(\frac{\theta}{2}\right)\right)^2 \qquad (2)$$

where k_B is Boltzmann's constant, T is temperature, η is the solvent viscosity (0.89 cP), d is the particle diameter, n is the index of refraction (1.33), θ is the is

the scattering angle (90°) and λ is the laser wavelength (633 nm). The method of cumulants uses a Taylor series expansion to express the exponential given in equation 1 as a polynomial, then fits the correlation function to this polynomial. The first coefficient, or moment (μ), is equal to Γ and used to determine the effective diameter. The second term is proportional to the variance of the weighted diffusion coefficient distribution (G(Γ)) and can be used to calculate a reduced second moment (μ_2') that is defined as the relative variance in this paper (in the referenced paper and conventional DLS it is referred to as the polydispersity).

$$\mu_2' = \text{polydispersity} = \frac{\mu_2}{\Gamma^2} = \frac{\mu_2}{\mu_1^2} \quad (3)$$

Further detail is given by Koppel [3]. Representative fitted data is shown in Figure S7 for compounds **4** and **5**.

Solution preparation: Prior to mixing, all solutions were filtered with 0.22 mm Millipore Millex ® GP filter units to eliminate dust interferences. Galectin-3 concentration was determined by measuring the absorbance at 280 nm using $\varepsilon^{1\%}$=6.1 ml/mg [4, 5]. Glycodendrimers **2**–**5** were prepared by dissolving the lyophilized powder in filtered Millipore water to a final concentration of 100 and 10 μM. Mannose-functionalized, generation 4 dendrimer was synthesized and characterized as described previously [6]. Varying amounts of glycodendrimer (0.14, 3.3 and 11.5 μM final concentration) were added to a galectin-3 solution (final concentration 31 μM) and incubated for 1 h at rt. The reported data is the

average of triplicate measurements on 1–6 samples. High relative variances were obtained for samples where the particle size was too small or too few particles were present to be detected (kcps < 50). High relative variances (>1) are characteristic of highly polydisperse samples and could not be fit well using the method of cumulants. This was observed at the highest concentration of dendrimer (no galectin-3 added).

Table S3: DLS signal and galectin-3 glycodendrimer aggregate diameter.

Compound number	[gal-3] (μM)	[dendrimer] (μM)	Effective dia. (nm)	Relative variance	Signal (kcps)[a]
	32	0	NA[b]	NA[b]	14 ± 6
**2**	0	0.14	NA[b]	NA[b]	5.5 ± 2.2
	0	11.5	NA[b]	NA[b]	23 ± 0.6
**3**	0	0.14	NA[b]	NA[b]	3.2 ± 0.8
	0	11.5	2.0 ± 0.4[c]	42 ± 34[d]	244 ± 63
**4**	0	0.14	NA[b]	NA[b]	3.8 ± 0.8
	0	11.5	6.2 ± 1.1[c]	69 ± 78[d]	167 ± 5
**5**	0	0.14	NA[b]	NA[b]	4.4 ± 0.3
	0	11.5	10.6 ± 2.2[c]	10.6 ± 2.5[d]	208 ± 8
**2**	31	0.14	NA[b]	NA[b]	28 ± 12
	31	3.3	364 ± 77	0.21 ± 0.05	60 ± 23
	31	11.5	781 ± 88	0.20 ± 0.04	135 ± 28
**3**	31	0.14	533 ± 72	0.16 ± 0.07	94 ± 24
	31	3.3	1744 ± 161	0.17 ± 0.06	282 ± 59
	31	11.5	1275 ± 377	0.19 ± 0.07	214 ± 134
**4**	31	0.14	1177 ± 81	0.19 ± 0.03	385 ± 26
	31	3.3	2027 ± 263	0.20 ± 0.10	246 ± 52
	31	11.5	871 ± 278	0.25 ± 0.04	292 ± 74
**5**	31	0.14	1616 ± 111	0.23 ± 0.09	271 ± 73
	31	3.3	2269 ± 266	0.23 ± 0.15	249 ± 65
	31	11.5	1316 ± 223	0.19 ± 0.08	252 ± 94
**3** + 1 mM lactose	31	0.14	NA[b]	NA[b]	14 ± 2
**4** + 1 mM lactose	31	0.14	NA[b]	NA[b]	17 ± 10
**5** + 1 mM lactose	31	0.14	NA[b]	NA[b]	25 ± 11
Mannose-functionalized G4	34	0.14	NA[b]	NA[b]	39 ± 11
	34(CRD)	0	NA[b]	NA[b]	5.4 ± 0.3
**5**	34(CRD)	0.14	NA[b]	NA[b]	10.4 ± 1.3
**4** + 60 μM lactose (27 μL)	31	0.14	NA[b]	NA[b]	47 ± 5.7
**4** + PBS (27 μL)	31	0.14	NA	NA	59 ± 5.5

[a]kcps = kilocounts per second.
[b]kcps < 50; particles too small to accurately fit using method of cumulants, NA = not applicable.
[c]Mean diameter of major peak (accounts for >99% of distribution population) from NNLS analysis (cumulant fit inadequate due to the bimodal nature of the sample as evident from high relative variances).
[d]Relative variance as reported from NNLS fit.

Fluorescence microscopy image calibration and sample preparation

Protocols for preparation of samples for fluorescence microscopy are provided below.

Table S4: Calibration equations for fluorescent standard microspheres.

Camera	Exposure time (ms)	Calibration curve equation[a]	R-squared value
Nikon	2	Y = 34.04x + 114.4	0.9658
	12	Y = 21.41x + 119.6	0.9894
	75	Y = 12.77x + 98.96	0.9986
	175	Y = 10.04x + 88.94	0.9992
Olympus	5[b]	Y = 15.94x + 355.8	1.0
	10[b]	Y = 14.19x + 309.8	0.9988
	40	Y = 10.79x + 164.9	0.9956

[a]Y represents particle diameter, and x is the number of pixels in the particle perimeter as quantitated by Pixcavator.
[b]190 nm standard could not be detected and was not included in the fit.

Table S5: DLS characterization of fluorescent microspheres.

Reported sample diameter (nm)	Measured effective diameter (DLS, nm)	Relative variance
190	196.6	0.005
520	511.8	0.025
1020	938.3	0.082
1900	1776.1	0.169

Sample preparation: Purified galectin-3 (3 mg/mL) was dialyzed (Spectrum Labs, MWCO 3500) for 2 h then overnight in 100 mM sodium bicarbonate buffer (pH 8.3). Roughly 0.2 mg AlexaFluor 488 succinimidyl ester (Molecular Probes) was dissolved in 20 μL DMSO and immediately added to 1 mL of the above galectin-3 solution. The reaction was rotated for 1 h at rt and was purified via dialysis (Spectrumlabs, MWCO 3500). The degree of labeling was determined according to the labeling protocol provided [7]. The solution of purified, Alexa-488 labeled galectin-3 was filtered and diluted to a final concentration of 31 μM.

Glycodendrimer–galectin samples were prepared as described for DLS (0.14 μM glycodendrimer, 1 h incubation at rt). To verify addition of dye molecule did not affect aggregation, particle sizes were verified by DLS prior to viewing. A 5 μL aliquot was mounted on a glass slide and covered with a coverslip. Average aggregate diameter and polydispersity (Table S6) were determined from data converted from pixels to diameter using the calibration curves.

Table S6: Aggregate diameter of A488gal-3 and glycodendrimers from fluorescent microscopy (FM) images and DLS.

Com-pound	n[a]	Exposure time (ms)	Avg diameter ± σ (FM, nm)	Relative variance (FM)	Effective diameter ± σ (DLS)	Relative variance (DLS)
**2**	59	135[b]	240 ± 47	0.20	Below detection	Below detection
**3**	223	12[b],10[c], 40[c]	703 ± 294	0.42	612 ± 27	0.19
**4**	137	12[b],10[c]	1070 ± 351	0.33	1243 ± 87	0.26
**5**	146	10[b], 5[c]	1788 ± 647	0.36	1700 ± 148	0.07

[a]Number of particles in image.
[b]Taken with Nikon Eclipse.
[c]Taken with Olympus BX-61.

Sample NMR spectra

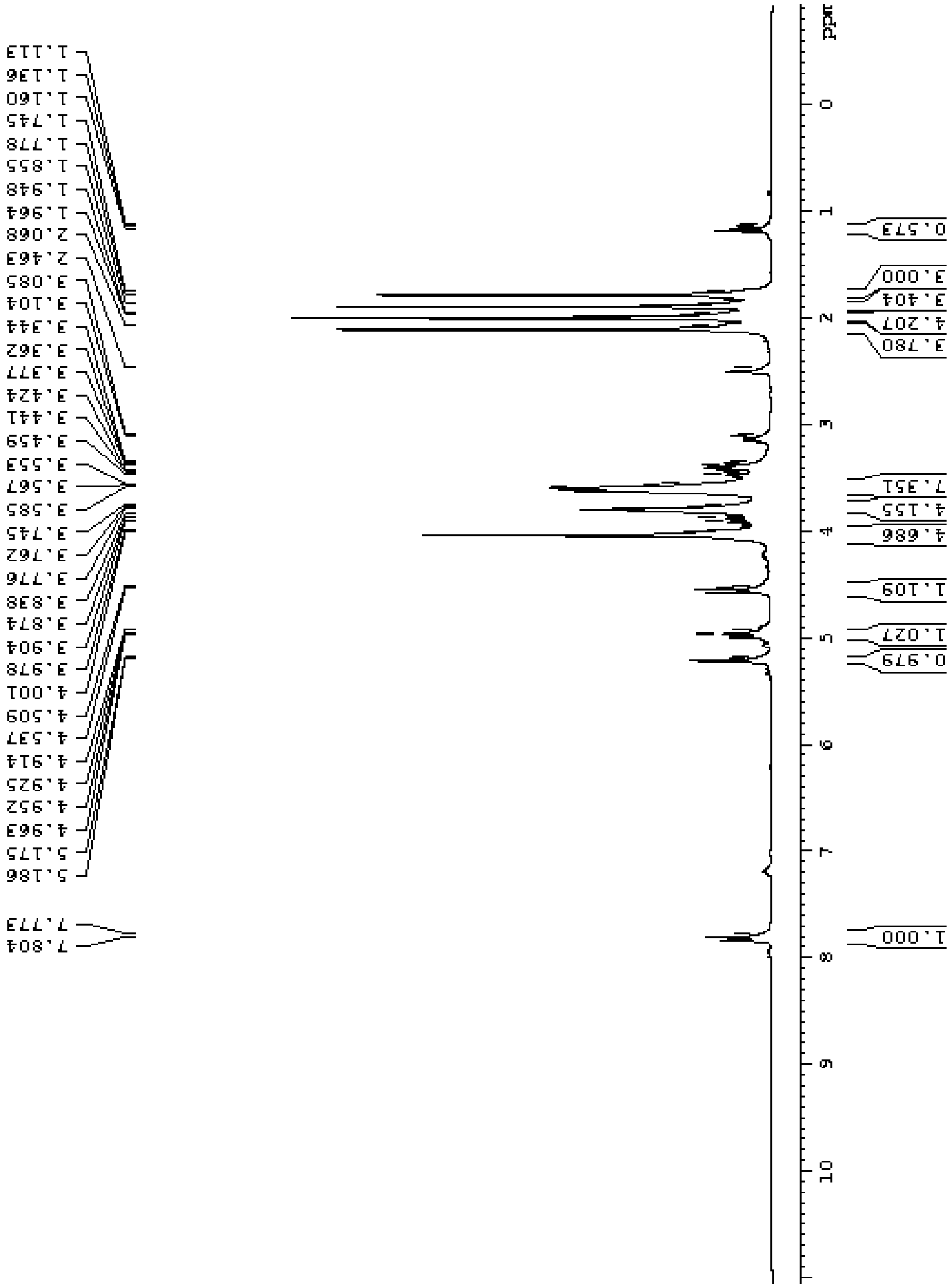

Figure S2: ^{1}H NMR spectrum (300 MHz, d_6-DMSO) of **1**.

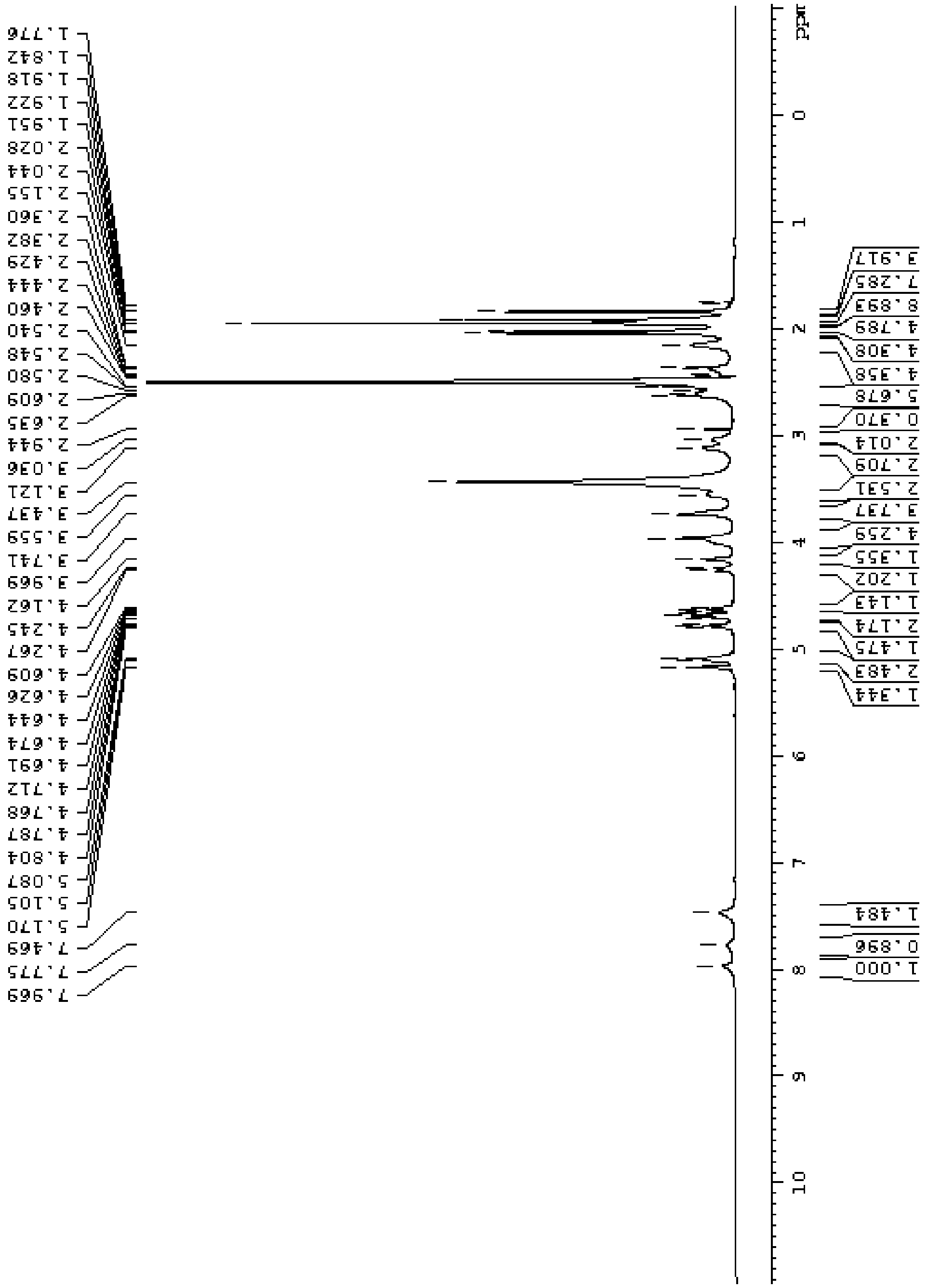

Figure S3: ^{1}H NMR spectrum (500 MHz, d_6-DMSO) of **3** (acetylated).

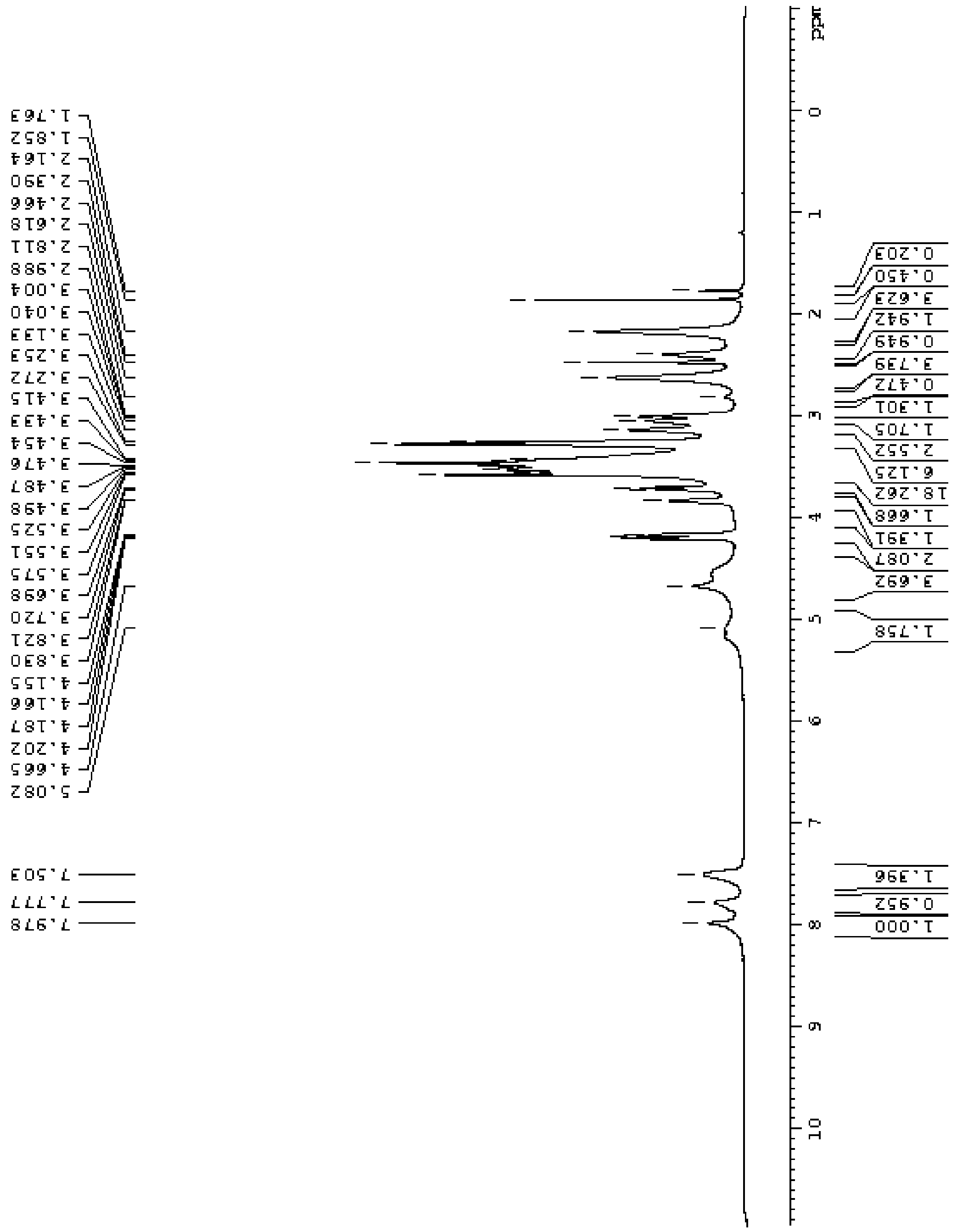

Figure S4: ^{1}H NMR spectrum (500 MHz, d_6-DMSO) of **3** (deacetylated).

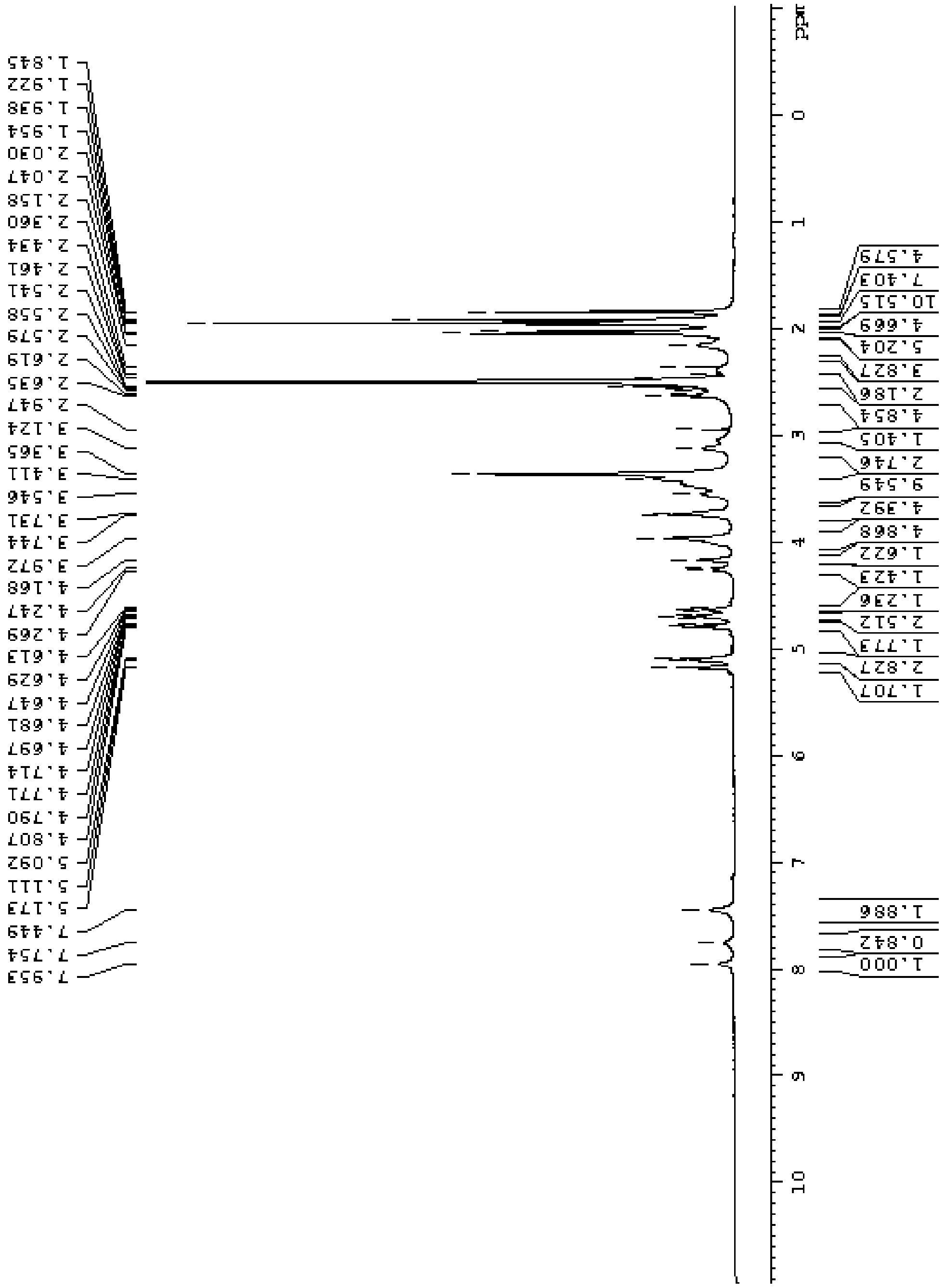

Figure S5: ^{1}H NMR spectrum (500 MHz, d_6-DMSO) of **4** (acetylated).

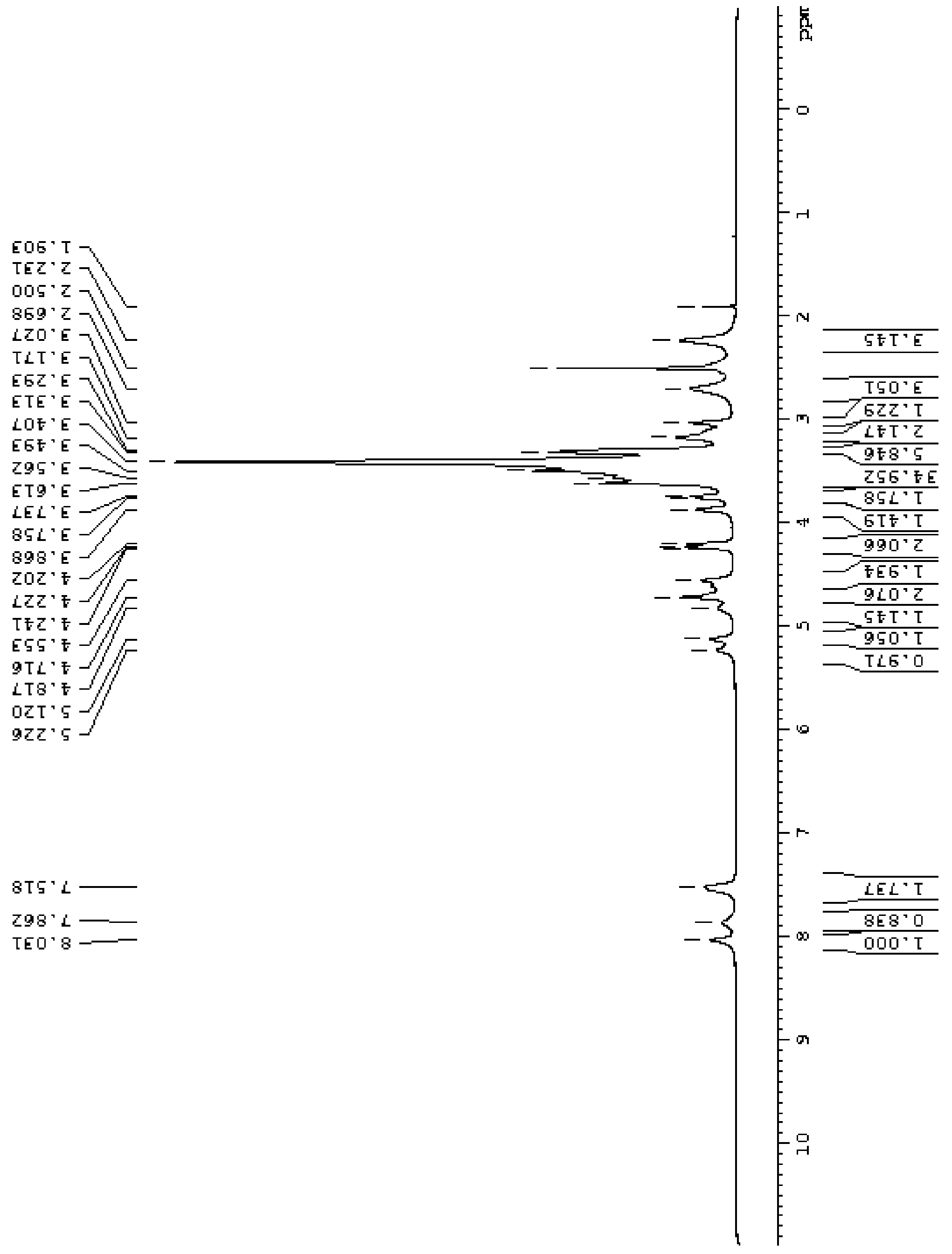

Figure S6: ^{1}H NMR spectrum (500 MHz, d_6-DMSO) of **4** (deacetylated).

Sample DLS correlation function

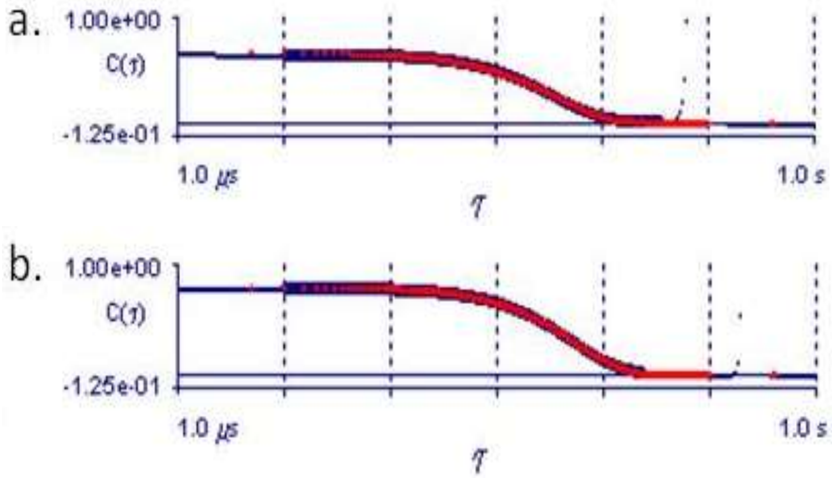

Figure S7: Representative DLS data (red) with quadratic cumulant fit (blue) for galectin-3 aggregates formed upon addition of glycodendrimers (a) **4** and (b) **5**.

Fluorescence microscopy calibration curves and exposure times

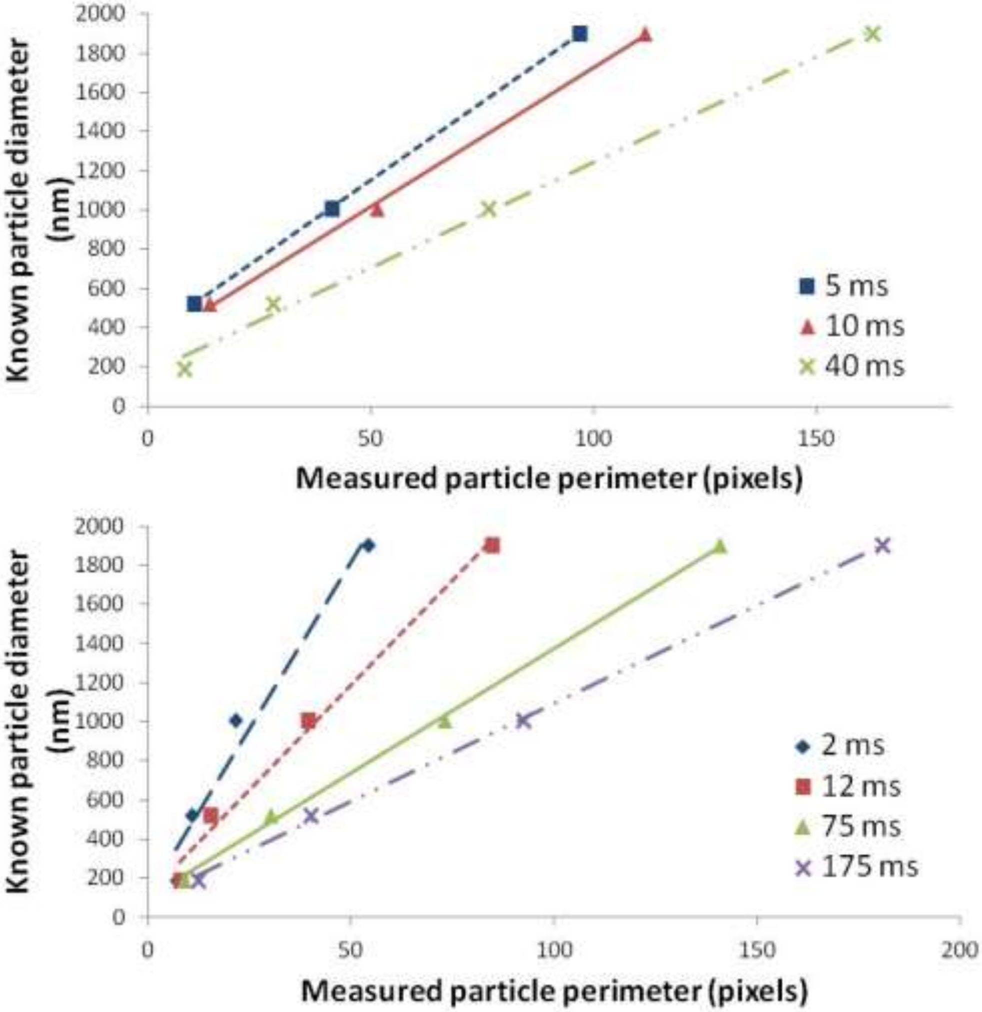

Figure S8: Calibration curves correlating pixel number to particle perimeter for (a) Nikon (b) and Olympus microscopes.

References

1. Nangia-Makker, P.; Balan, V.; Raz, A. *Meth. Mol. Biol.*, **2012**, 251-266.

2. Ahmad, N.; Gabius, H. J.; Andre, S.; Kaltner, H.; Sabesan, S.; Roy, R.; Liu, B. C.; Macaluso, F.; Brewer, C. F. *J. Biol. Chem.,* **2004**, *279*.

3. Koppel, D. E. *J. Chem. Phys.* ***1972***, *57*, 4814.

4. Smith, P. K.; Krohn, R. I.; Hermanson, G. T.; Mallia, A. K.; Gartner, F. H.; Provenzano, M. D.; Fujimoto, E. K.; Goeke, N. M.; Olson, B. J.; Klenk, D. C., *Anal. Biochem.* **1985,** *150*, 76-85.

5. Ahmad, N.; Gabius, H. J.; Kaltner, H.; Andre, S.; Kuwabara, I.; Liu, F. T.; Oscarson, S.; Norberg, T.; Brewer, C. F. *Canadian J. Chem.-Revue Canadienne De Chimie,* **2002,** *80*, 1096-1104.

6. Wolfenden, M. L.; Cloninger, M. J. *Bioconj. Chem,.* **2006,** *17*, 958-966.

7. *Molecular Probes Handbook, A Guide to Fluorescent Probes and Labeling Technologies*. 11th ed.
